# A scoping review of the impact of eco-distress and coping with distress on the mental health experiences of climate scientists

**DOI:** 10.3389/fpsyg.2024.1351428

**Published:** 2024-10-21

**Authors:** Luis Calabria, Elizabeth Marks

**Affiliations:** ^1^Department of Psychology, The University of Bath, Bath, United Kingdom; ^2^Bath Centre for Mindfulness and Community, Bath, United Kingdom

**Keywords:** eco-distress, eco-anxiety, climate change, climate scientists, psychology

## Abstract

**Introduction:**

In the face of a future predicted to be one defined by an increase in the Earth’s surface temperature, and the associated extreme weather events, distressing emotional responses are understandable. Climate scientists comprise a unique group, in that they are deeply and consistently faced with the current reality, and consequences of, climate change. ‘Ecological distress’, a term to describe climate-related emotional experiences, can include feelings of grief, anxiety, and hopelessness proportionate to the existential threat of climate change.

**Methods:**

This review sought to scope the current literature into how ecological distress is experienced by this population, factors that mitigate or exacerbate these experiences, and the coping strategies that are used. This review used Interpretive Content Analysis to code the data and employed a narrative synthesis of the findings.

**Results:**

This paper demonstrates the significant emotional impacts associated with working as a climate scientist, along with an associated set of coping strategies. Climate scientists share experiences of emotional distress, involving both intra- and interpersonal processes, highlighting how people working in this profession may be vulnerable to unique pressures and psychological burdens related to their work.

**Discussion:**

Climate scientists have an essential role to play in helping society and policy makers understand the implications of climate change and identifying the most useful responses. As such, it is integral that the psychological wellbeing of this group is understood and cared for. This review is the first step in synthesising current knowledge, in preparation for developing helpful methods and strategies for keeping our climate scientists well, motivated, and engaged.

## Introduction

As greenhouse gas emissions increase, it is predicted that there is a greater than 50% chance that the surface temperature of the Earth will increase by 1.5°C by 2040 (Pörtner et al., 2022). Recent ‘tipping points’ in the climate crisis include signs of the collapse of the Gulf Stream (Boers, 2021), the Amazon Rainforest becoming a source of carbon emissions rather than a sink (Gatti et al., 2021), the release of methane from the Siberian permafrost following the 2020 heatwave (Froitzheim et al., 2021), and the heatwave and fires across Europe in July and August 2022. Together, these data provide an uncertain picture of the future of human life on Earth, and how a global community might mitigate against the changes following these events.

The United Kingdom government website defines a climate scientist as an individual who ‘stud[ies] changes in the Earth’s climate over time and how they might affect the planet in the future’ (National Careers Service, 2023). As the lens through which the impacts of climate change on the planet widens, the definition will likely include a growing range of disciplines and individuals.

Climate scientists are at the forefront of gathering information and understanding the risks and implications of climate related projections. Climate scientists also play an important role in helping society and policy makers understand the implications of climate change, and identifying what responses can reduce, mitigate, and adapt to the impacts of climate change. Repeated exposure to current and future threats may have significant psychological consequences. This review will seek to scope out the current literature to best understand the emotional and psychological experiences of climate scientists.

Eco-distress refers to the broad range of psychological burdens that arise in response to the climate and ecological emergencies: ‘*the generalised sense that the ecological foundations of existence are in the process of collapse*’ (Albrecht, 2012, p.205). The term eco-distress will be used throughout this paper except when referring to studies which have examined specific experiences such as ‘eco-anxiety’ or ecological grief. Climate scientists have not, traditionally, been studied or thought of as an at risk group. As such, the theoretical underpinnings of this review are based on the eco-distress experiences of other groups. A high proportion of people have expressed distress in response to the realities of the climate and ecological emergencies (i.e., ecological/eco-distress) (Hickman et al., 2021). This is characterised by a range of painful, emotional and cognitive responses to the growing awareness of the very real threats of climate change including eco-anger, eco-anxiety, and eco-depression (Stanley et al., 2021). Climate scientists are in regular and close proximity to some of the most threatening and cutting-edge information about climate change. As a group, they may be particularly vulnerable to ecological distress, as they are routinely exposed to information that keeps the threat of climate and environmental crises at the forefront of their minds. Unlike the young people in Hickman et al.’s (2021) study, climate scientists operate from a position of relative power. This powerful position may, in fact, be protective for climate scientists.

The mental health impacts of ‘ecological distress’ are unclear, with the definition being currently broad enough to capture a wide range of emotional responses. Additionally, researchers propose that it is a rational, and potentially constructive, response (Verplanken and Roy, 2013; Verplanken et al., 2020). Nevertheless, awareness of the threats posed by the climate and ecological crises, coupled with the lack of action by those in authority (i.e., governments), may be a chronic psychological stressor.

### The relevance of intrapersonal processes in eco-distress

‘Ecological distress’, as described above is a source of psychological pain, but is not a mental illness (Verplanken et al., 2020). One of the main arguments in support of this is that eco-distress arises from an accurate and rational interpretation of the existential threat humanity is facing, and which is set to get worse (Clayton, 2020; Verplanken et al., 2020). Cognitive behavioural theory posits that psychological distress arises from the *meaning* individuals make of their experiences, rather than the experience itself: ‘events and other people do not make us ‘feel good’ or ‘feel bad’; we do it to ourselves, cognitively’ (DiGiusseppe et al., 2014, p.21). How one thinks about a situation impacts how one feels and acs, and in many cases of psychological distress, the meaning an individual may create can be unrealistically negative and catastrophic, leading to a situation where their belief about a threat is far more distressing than the most likely outcome. In contrast, the reality of climate change is that the threats are catastrophic, and many are already happening. Accurate thoughts about the current and predicted future impacts of climate change thus, understandably, lead to feeling paralysed and hopeless.

A recent review (Maiella et al., 2020) found that greater psychological proximity to climate change was associated with increased pro-environmental behaviours. As such, ecological distress can be associated with positive aspects of the self, including a strong sense of environmental identity (Verplanken and Roy, 2013; Verplanken et al., 2020), as well as with adaptive behaviours which aim to mitigate and reduce the threat (Whitmarsh et al., 2022). Thus ecological distress appears not be characterised by the types of unhelpful beliefs, assumptions, and core beliefs which are commonly linked to mental illness such as depression (e.g., Dozois and Beck, 2008). Instead, ‘ecological distress’ is likely to arise in individuals (such as climate scientists) who have core beliefs and values relating to the health and wellbeing of the planet, other species and other people. These values and beliefs are combined with a realistic interpretation of the threat to life on Earth that the climate and ecological crises represent, and a commitment to acting in a way that will mitigate this threat.

Despite ‘ecological distress’ being reported by climate experts, some research has indicated that there may be a tendency to avoid exploring or sharing such emotions with others, for example, by keeping them ‘in a box in my head’ (Andrews, 2017, p.10). This can be described as an emotionally avoidant coping strategy, and is likely to have advantages and disadvantages for the individual and society more broadly. The degree to which it is used, however, and its consequences are not yet well explored. This approach could, for example act as a protective mechanism to allow continued engagement with the science. Alternatively, as described by the Limited-Resource Model of Self-Regulation, ongoing attempts to regulate emotions in this way could have the unintended consequence of depleting one’s resources, making it harder to do again in the future (Vohs et al., 2011), potentially leading to burn-out, or even increased risk of earlier death (e.g., Chapman et al., 2013).

Despite being regularly exposed to information likely to fuel their distress, climate scientists, like others reporting ecological distress, have limited space to air their concerns. This can be understood as a ‘socially constructed silence’ (Fivush, 2010), where emotional and cognitive responses to climate change are not allowed to be talked about in many social spaces. It can be seen in the reports of people being ignored or dismissed when they try to talk about climate change (Hickman et al., 2021). This limits both individual emotional expression, and social forms of expression such as adaptive and community-based coping, where people find solidarity with others who share their experiences (Mah et al., 2020). For climate scientists regularly exposed to highly distressing information about the climate crisis, they are likely to experience painful emotional and cognitive responses. The impact of this on their functioning and health is as unclear. Furthermore, as many climate scientists centre their professional lives around pro-environmental action, their experience of managing such distress may be different from other populations who potentially have less ability to engage in such action, when considering the relationships between pro-environmental behaviours and ecological distress (Clayton, 2020; Verplanken et al., 2020).

### The relevance of interpersonal processes in eco-distress

There are important relational aspects to eco-distress, since it is both caused by humans and since the solutions depend on actions being taken by other people. Climate scientists assume a unique position in that their exposure to climate realities comes from a deep scientific knowledge of the field. In addition to the direct psychological burdens of this, the way in which climate scientists’ findings are reported by the media, and how such findings are received by the public, can bring its own challenges, with many scientists having experiences of being disbelieved, publicly criticised, and even vilified (Bowe et al., 2014). Such negative reactions and conflict with the public and media may exact an emotional toll, but this has not been well investigated. Experiences of being disbelieved by others is known to cause emotional distress in other populations, such as those with chronic pain (Newton et al., 2013). Furthermore, exposure to vilification and public threats may be linked to greater psychological distress, for example in judicial officers where threat frequency and threat concern were each correlated with higher scores on measures of PTSD (O'Sullivan et al., 2022).

The mixed nature of public opinion on the climate and ecological crises is demonstrated by recent polls indicating moderately high levels of ‘alarm’ or ‘concern’ about climate change, in 67% of people in the United Kingdom, and 58% in the United States (Leiserowitz et al., 2021). This, however, leaves over a third of people not particularly concerned, while only about half (55% United Kingdom and 40% United States) of respondents believe that climate change was ‘caused mostly by humans’ (Leiserowitz et al., 2021). Phillips et al. (2018) reported that while 93% of British adults believed climate change was happening, only 36% thought it was entirely/mainly anthropogenic, and only 25% were ‘extremely/very worried’. Thus public opinion still does not accurately reflect scientific consensus published by the International Panel on Climate Change (IPCC). So, despite climate scientists raising the alarm, their warnings are frequently ignored, disavowed and even disbelieved.

Väliverronen and Saikkonnen (2021) report an increase in ‘disturbing feedback’ towards scientists from 2015 to 2017 and suggest that as political and organisational control on science increases, scientists may self-censor more (e.g., remaining silent for fear of damage to reputation of career). If they feel attacked or undermined publicly, climate scientists may become reluctant to publish future research, or engage in self-censorship. This could lead to society and policy makers being less informed, and could also reinforce painful feelings of guilt, shame, and self-doubt in climate scientists who might feel that they are not doing enough.

### Moral distress and moral injury

One of the relational aspects of eco-distress that may be particularly important is the experience of moral distress and injury. Moral distress can arise when ‘institutional constraints make it nearly impossible to pursue the right action’ (Jameton, 1984, p.6). The experience of climate anxiety in children and young people has been likened to moral distress, as their perception of government inaction on the issue (underpinned by climate science) has been experienced as a betrayal, which in turn is associated with greater climate anxiety (Hickman et al., 2021). Moral injury (a more chronic and distressing psychological experience) can occur when an individual loses confidence in ‘one’s own or others’ motivation or capacity to behave in a just and ethical manner’ (Drescher et al., 2011, p. 9). It may arise when one either sees or engages in actions of commission or omission that violate moral or core beliefs (Griffin et al., 2019; Evans et al., 2020). Molendjik (2018) suggests that perceived societal misrecognition (which could include the misrepresentation or harsh public criticism of climate scientists) can be a morally injurious event. Such experiences can lead to feelings of remorse, betrayal, and separation from others. Moral distress and moral injury have been experienced by NHS staff during the COVID-19 pandemic (Lamb et al., 2021), and by journalists covering the migrant crisis in 2015 (Feinstein et al., 2018), where people considered themselves to have acted in violation with their moral codes. High levels of moral injury have implications for mental health and were significantly associated with anxiety, depression, alcohol misuse, and PTSD in healthcare workers working during the COVID-19 pandemic (Lamb et al., 2021). It follows that these outcomes negatively impact the ability of healthcare workers to thrive in their job. In the same way that it is essential to protect the mental health of healthcare staff in a pandemic, it is important to protect the mental health of climate scientists whose contribution to society is equally valuable.

Definitions of moral injury include feelings of powerlessness (Morley et al., 2019). It could be that climate scientists, as leaders in their field, are perceived as powerful, and so presumed to be not susceptible to moral injury. Nevertheless, evidence suggests that scientists can still experience dismissal and disavowal (Väliverronen and Saikkonen, 2021). How might moral injury present in a group seen as having a position of power? Similarly, moral injury in health care workers, for example, involves close proximity to the impacts of acts of commission or omission (Lamb et al., 2021). How might moral injury present in a group with greater distance from the direct impacts of their actions? This review will aim to scope the existing literature, and develop an understanding of the morally injurious experiences of a group seen as powerful, and somewhat distal from the threat they are studying.

As global temperatures continue to rise, the role of climate scientists becomes ever more important in helping us to both understand and positively engage with the impacts of climate change. Yet they face a range of professional related stressors including persistent exposure to negative climate-related information, negative responses from society to the science they publish, ranging from ignoring and dismissal (Leiserowitz et al., 2021), to anger and misrepresentation (Bowe et al., 2014). There also appears to be a lack of space in which they can discuss and explore such psychological burdens, all of which are likely to impact their mental health and wellbeing. The primary aim of this review is to scope the existing evidence base, to better understand the psychological impacts upon climate scientists. The secondary aim is to identify what future research is required, and how to best support this group of professionals so they can continue to contribute science to the public, businesses, and policy makers that is so essential to our own, and our planet’s, survival.

## Current study

The current study aimed to scope the literature describing the psychological experience and mental health of climate scientists, to explore the extent and characteristics of existing studies, summarise findings from studies of heterogeneous methodologies and identify gaps in the literature, by answering the following questions:

1. What evidence exists regarding climate scientists’ mental health experiences in relation to climate change and their work?
2. What intrapersonal processes are related to climate scientists’ experiences of climate change and their work?
3. What interpersonal processes are related to climate scientists’ experiences of climate change and their work?
4. What mitigates and what exacerbates these experiences?

## Methods

### Search methods

This Experiential (Qualitative) Review (Munn et al., 2018), followed a Population, Phenomena of Interest, Context (PICo) format (Lockwood et al., 2015). A systematic, electronic search strategy was carried out to identify peer-reviewed articles, exploring the mental health experiences of Climate Scientists as follows:

- Population: Climate Scientists.
- Phenomena of Interest: Mental Health Impact (Depression, anxiety, PTSD, Low Mood, Hopelessness, Helplessness, and Anger).
- Context: Societal narratives about climate change.

Table 1 details the keywords used for the review of the literature.

**Table 1:** Search terms.

1	‘climate scientist*’ or ‘{climate scientist}’ OR ‘climate researcher*’ or ‘{climate researcher}’ OR ‘climate expert*’ or ‘{climate expert}’ OR ‘sustainability professional’. *The appropriate format was used depending on which database is being searched* (i.e., ‘*’ for Web of Science and Scopus, and ‘{}’ for International Bibliography of the Social Sciences).
	*AND*
2	‘climate change’ OR ‘global warming’
	*AND*
3	‘mental health’ OR ‘mental illness’ OR ‘emotion*’ OR ‘depress*’ OR ‘stress’ OR ‘distress’ OR ‘grief’ OR ‘mood’ OR ‘anxi*’ OR ‘PTSD’ OR ‘posttraumatic stress’ OR ‘post-traumatic stress’ OR ‘traumatic stress’ OR ‘eco-anxiety’ OR ‘ecoanxiety’ OR ‘eco anxiety’ OR ‘climate anxiety’ OR ‘climate-anxiety’ OR ‘hopeless*’ OR ‘helpless*’ OR ‘anger’ OR ‘resilien*’ OR ‘psych*’.

The search was initially conducted on 23 August 2022, and repeated on 19 May 2024 to update the findings, on the following databases: PsychInfo, Pubmed, Web of Science, Scopus, and International Bibliography of the Social Sciences (IBSS). These databases were selected after consultation with the University librarian. Given the multidisciplinary nature of this review, these databases were deemed most appropriate. Given the relative novelty of this field as an area of research, it was decided that broad search terms would be used. Arksey and O’Malley’s (2005, p.23) framework allows for exploring ‘reference lists, hand-searching of key journals, existing networks, relevant organisations and conferences’. It was decided, however, to search in a more systematic way to improve transparency, replicability, and a more methodologically sound approach. Table 2 details the inclusion and exclusion criteria. Titles and abstracts were then reviewed, in order to ascertain the appropriateness of the papers to be included. An independent research assistant screened 10% of the abstracts screened by the lead researcher. No additional studies were highlighted for inclusion. The lead researcher then screened the remaining full texts against the inclusion and exclusion criteria.

**Table 2:** Inclusion/exclusion criteria.

Must be related to ‘Climate Scientists’ as a population. Participants had to be scientists working in fields directly studying climate change. Participants considered ‘sustainability professionals’ in a wider context were excluded as this is a less protected term, and would have included a much broader population which would have impacted upon the specificity of the findings.
The review included quantitative, qualitative, and mixed methods designs.
The review excluded books, and book chapters.
Studies were not to be excluded if they had not been translated into English. Translations would be sought from the original authors.
Grey literature (e.g., policy statements, and theses and dissertations) was not excluded from the studies.
Literature was not excluded based on publication date.

### Analysis

The research team in this review adopted a critical realist ontological stance, where reality is considered subjective based on the different phenomenological experiences of each individual. Simultaneous explanation and interpretation of data allows researchers to explore complex and interlinked realities (Cuthbertson et al., 2020). Epistemologically, critical realism advocates for the pursuit of developing models and structures to progress knowledge (Cuthbertson et al., 2020). As such, studies involving hundreds of participants (e.g., Jovarauskaite and Böhm, 2020) were included alongside commentary and reflective pieces (e.g., Reay, 2018). All of these phenomenological experiences were deemed equally worthy, and provided data that might usefully inform models of knowledge around climate scientists’ distress. A scoping review was undertaken in a way that permits the synthesis and analysis of a wide range of research and non-research material, i.e., including the ‘grey literature’ with the aim of developing greater conceptual clarity about a specific topic or field of evidence (Davis et al., 2009) such as this one. Grey literature includes literature produced in government, academia, and business but is not published by commercial publishers (Paez, 2017). While this review searched databases that include grey literature (i.e., Scopus and Web of Science), none was identified as relevant to this scoping reviewing. Commentary and reflective pieces published in peer-reviewed commercial journals were included, as they were clearly pertinent to the topic although they did not report raw data as such.

Arksey and O’Malley’s (2005) framework for scoping reviews requires that the data from selected studies are ‘charted’. This involves synthesising and organising the data into key sub-headings (see Appendix A). The research questions were used to organise the sub-headings during the charting process. Scoping reviews involve a systematic approach to data collection but require an iterative process in knowledge synthesis (O’Brien et al., 2016), guided by the original research questions. This allows for flexibility in exploring this novel topic, but has implications for understanding the conclusions drawn, as interpretation uses a less rigid framework than in other types of review. The charting process in this review was completed by the researcher and an independent research assistant. Additional review of the charted data was completed by the research supervisor.

No quality assessment was conducted, as it is not a requirement of scoping review methodology (Arksey and O’Malley, 2005). Rather, the scoping review aimed to follow the methodological approach which encourages the synthesis of a wide range of research and non-research data, based on the critical realist epistemological approach that advocates for exploration of complex and interlinked realities. From this stance, both a randomised control trial and a single case reflective piece offer similarly valuable data to any model or structure being developed, and thus grey literature can add important information to the review, allowing concepts to be better developed, explored and refined. This was particularly important for the current topic which is nascent and for which there is limited published research.

In this review, Interpretive Content Analysis was used to code the data, which involved the researcher drawing interpretations and insights to generate codes and themes that goes beyond literal codes based purely on semantic content. Unlike traditional Content Analysis, Interpretive Content Analysis is not constrained by strict coding rules and allows researchers to account for wider contextual factors in the analysis (Ahuvia, 2001). Interpretive Content Analysis sacrifices inter-rater reliability for the opportunity to code the data in the context of the whole research paper, and note the subtle links and meanings in the data (Ahuvia, 2001). This fits the critical realist stance of the researchers in this review, as phenomenology is accounted for, and interpretation is facilitated. Given the limited literature on this topic, and the high levels of qualitative research in this area, Interpretive Content Analysis was deemed the most appropriate methodology. The coding and charting process was completed by two members of the research team, separately, who then met to compare their codes and agree the most compelling interpretation of the data (Ahuvia, 2001).

## Results

**Figure 1:**
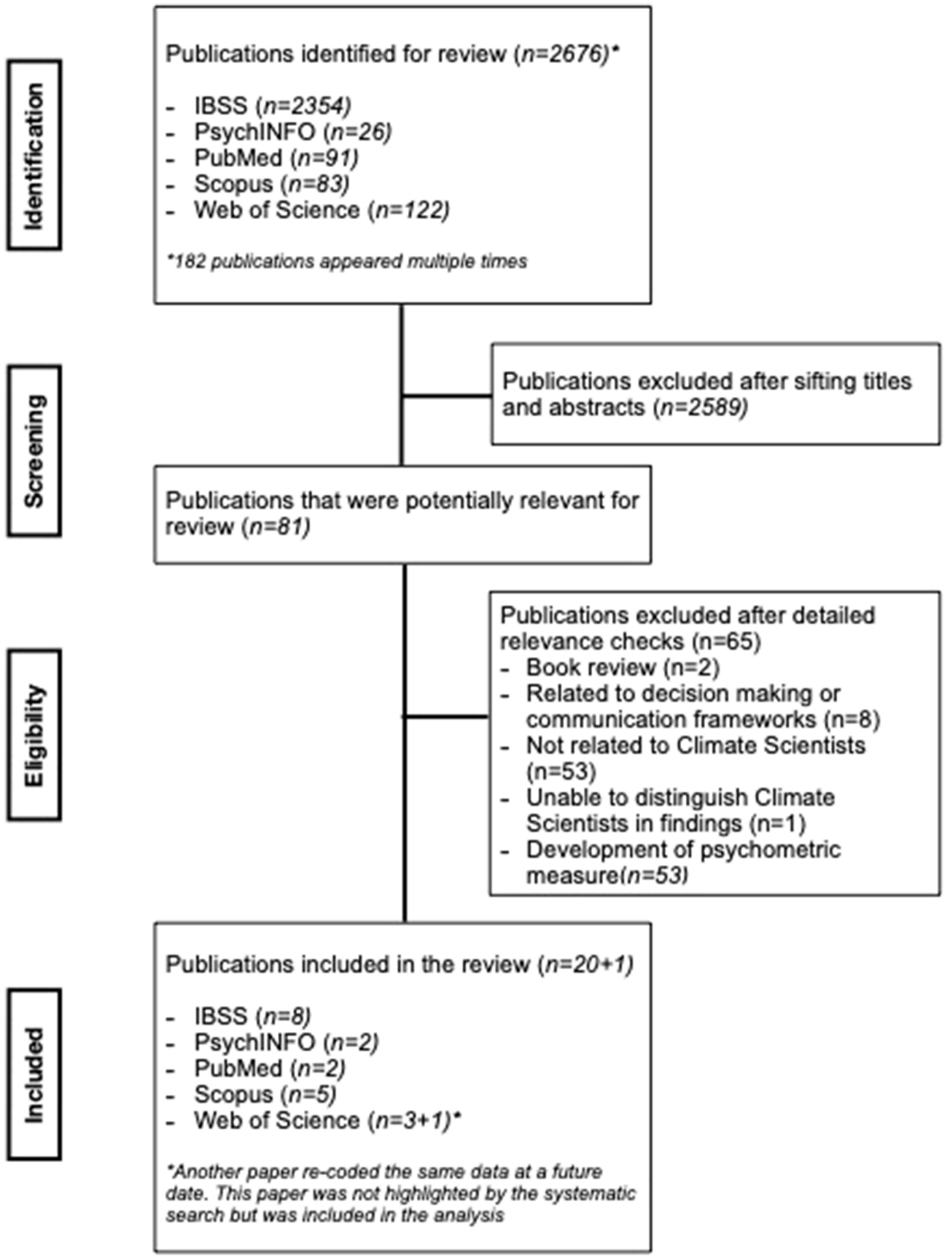
Systematic search flow diagram.

The search generated 2,676 references, from which 87 potentially relevant abstracts were identified. After a review of the full text, 20 publications remained. Table 3 presents details of the 17 included publications. A further reference was sourced after hand-searching reference lists, as it was a re-coding of the data in one of the included papers. Figure 1 presents the flow diagram for the selection and exclusion process of included articles.

**Table 3:** Details of publications included in the review.

Author(s)	Year	Title	Design	Participants
Beck	2012	Between tribalism and trust: the IPCC Under the ‘Public Microscope’.	Review of how the Intergovernmental Panel on Climate Change (IPCC) has dealt with increased public scrutiny since, so called, ‘Climategate’.	N/A
Bodenhorn	2013	Of time and forest fires, or what are scientists for anyway?	Review of the relationship between evidence, modelling, and prediction.	N/A
Campbell-Lendrum and Bertollini	2010	Science, media, and public perception: implications for climate and health policies.	Editorial	N/A
Clayton	2018	Mental health risk and resilience amongst climate scientists.	Secondary data analysis of frequency of emotions referenced in the ‘is this how you feel’ project.	43 responses analysed.
Cologna and Siegrist	2020	The role of trust for climate change mitigation and adaptation behaviour: a meta-analysis.	Meta-analysis	46 articles included.
Duggan et al.	2021	Climate emotions: it is ok to feel the way you do.	Secondary data analysis of frequency of emotions referenced in the ‘is this how you feel’ project.	73 letters
Finnerty et al.	2024	Scientists’ identities shape engagement with environment activism.	Mixed methods design across a number of countries. Aimed at exploring association between scientist identity and science-activism relationship.	329 scientists from 41 countries
Hayhoe	2021	Talk, act, hope.	Secondary data analysis of frequency of emotions referenced in the ‘is this how you feel’ project.	43 participants
Head and Harada	2017	Keeping the heart a long way from the brain: the emotional labour of climate scientists.	Interviews conducted with climate scientists.	13 participants
Herman et al.	2018	Alerters, critics, and objectivists: researchers in Austrian newspaper coverage of climate change.	Newspaper articles contributed to by climate scientists were analysed. Coding and interpretation of information, and ‘rhetorical devices’ was used to ascertain what ‘type’ of scientist was contributing (i.e., alerter, critic, objectivist).	‘34 different researchers presented their perspectives on climate change issues in the analysed interviews and guest commentaries’.
Jaspal et al.	2012	Contesting science by appealing to its norms: readers discuss climate science in the daily mail.	Discourse analysis of reader comments on articles pertaining to climate science and/or climate scientists.	1,907 reader comments included in the analysis.
Jovarauskaite and Bohm	2020	The emotional engagement of climate experts is related to their climate change perceptions and coping strategies.	Participants completed online questionnaires regarding their views and opinions about climate change.	215 participants
Light et al.	2021	Clouding climate science: comparative network and text analysis of consensus and anti-consensus scientist.	Text analysis of anti-consensus and consensus climate scientists.	7,354 articles written by 57 anti-consensus scientists, and 270 consensus scientists.
Miner	2023	I’m a Climate Scientist. Here’s How I’m handling climate grief.	Personal reflection on her own lived experience of climate change as a climate scientist.	N/A
Nicolaisen	2022	A state of emergency or business as usual in climate science communication? A three-dimensional perspective on the role perceptions of climate scientists, climate journalists, and citizens.	Focus group exploring the perceptions on the roles of climate scientists, climate journalists, and citizens.	15 focus groups with 26 Danish climate scientists, 24 climate journalists, and 26 citizens.
Reay	2018	How I stave off despair as a climate scientist.	Personal reflection on his own lived experience of climate change as a climate scientist.	N/A
Renouf	2021	Making sense of climate change—the lived experience of experts.	This is the first stage of a wider research project. This part of the research draws on 16 qualitative interviews with climate scientists to explore the meaning that climate scientists give to climate change through their lived experience.	Thirteen of the 16 participants had contributed to IPCC reports.
Spies	2017	The science and politics of climate change	Review attempting to unravel the science from the politics, describe typical emotional responses, and discuss the importance of, and barriers to, achieving an international agreement on reducing emissions.	N/A
Tangey	2018	Between conflation and denial: the politics of climate expertise in Australia.	This paper uses a review methodology to examine climate experts’ rhetorical tactics through the eyes of conservative policymakers.	N/A
Tollefson	2021	Top climate scientists are sceptical that nations can rein in global warming.	Feature article, analysing IPCC survey data.	92 out 233 scientists responded.
Wang et al.	2018	Emotions predict policy support: why it matters how people feel about climate change.	The first study examined responses from climate scientists using data collected externally by the ‘Is this how you feel’ project, in addition to responses from a new survey conducted by the current authors, asking students to report their feelings about climate change.	Scientist data (*n* = 44) Students (*n* = 94)

### Article characteristics

#### Year and location of studies

All included articles were published between 2010 and 2024. Seven of the studies were conducted in Australia, four in each of the United Kingdom and the United States, two in Switzerland, and one in each of Austria, Lithuania, Denmark, and Germany. All countries present in the data for this review were ‘WEIRD’ (Western Educated Industrialised Rich Democratic) countries.

#### Article type

Seven articles (39%) included original data. Four (17%) employed quantitative methods, three (13%) used qualitative methods, and two used a mixed methods design (7%). Four articles (17%) used secondary data for their analysis. Seven articles (35%) were without original data and were categorised as a commentary, editorial, or review.

Three of the papers (i.e., Clayton, 2018; Duggan et al., 2021; Wang et al., 2018) analysed data gathered from the same ‘Is This How You Feel Project’. Firstly, this speaks to the poverty of data, currently, exploring the psychological experiences of climate scientists. Given the critical realist epistemological stance of the research team, it was decided to include all three papers in the review. Each of the aforementioned papers will draw distinct conclusions based on the researchers’ own phenomenological experience with the data. Additionally, the temporal differences in when the data were analysed (i.e., Clayton, 2018; Duggan et al., 2021) mean that the dataset will have developed.

## Findings and interpretation

A narrative synthesis was used to bring together the findings in this review. Narrative syntheses allow for the exploration of relationships within the data, as well as facilitating theory building based on the data (Popay et al., 2006). In this review, the findings and interpretations are presented together. This is to allow for the presentation of a clearer narrative as the data are explored in relation to the rest of the data, as well as existing psychological theories and models. Research questions scaffolded the subsequent narrative about how emotions related to climate change are experienced by climate scientists, the intra- and interpersonal processes that contribute to this, and, within this, factors that exacerbate and/or mitigate these experiences.

### Climate scientists’ psychological experiences

#### Painful emotions and distress

Seven of the papers commented on the emotional responses of climate scientists to climate change. None specifically referenced mental health problems, and only notes issues using terminology associated with ecological distress (i.e., climate grief; Miner, 2023). There were, however, frequent expressions of painful emotions in response to climate change which align well with such experiences. In one survey of 92 IPCC scientists, 61% reported feelings of anxiety, grief, or distress as a result of climate change, with 21% reporting that these feelings were frequent (Tollefson, 2021). The same survey explored the behavioural impacts of these emotions, ranging from choosing where to live (41% of respondents), to changes to lifestyles (21%) to decisions about having children (17%).

Clayton (2018) analysed 43 letters written by climate scientists in the ‘Is This How You Feel’ project, and reported that ‘frustration’, ‘concern’, and ‘sadness’ (noted 13, 11, and 9 times, respectively) were commonly reported emotional responses, but that so was ‘hopefulness’ (noted 11 times). Similarly, Wang et al.’s (2018) analysis of data from the same project noted that ‘frustration’ was reported by 16 of the 44 scientists who responded. This is in line with reports of ecological distress in other populations, where emotions of different valences are present within individuals (or indeed the same individual) concerned about climate change (Hickman et al., 2021; Pihkala, 2022).

Duggan et al. (2021) re-analysed this data at a later point, after more contributions. They reported that ‘afraid’ and ‘angry’ were the most commonly referenced emotions (noted 69 and 49 times, respectively). Throughout the duration of the project, some scientists contributed twice, reflecting on their own emotional experiences, and any changes or developments in their experiences. The frequency of emotions like ‘afraid’ or ‘angry’ being referenced increased in second time contributors, indicating that such emotions either become more common with time, or that one’s awareness of, and ability to report, them increases with time. Renouf (2021), interviewed 16 climate scientists, reporting ‘anger’, ‘sadness’, and ‘rage’. The timing of these interviews was concurrent to the 2019 Australian bushfires, which may have impacted responses. This represents the context in which humanity is living. As extreme heat, fires, droughts, storms, and flooding continue to increase in frequency and intensity across the world, so the experiences of climate scientists will reflect the nature of the changing (and worsening) effects of the climate crisis.

Jovarauskaite and Böhm (2020) explored the emotional responses to climate change of 215 Lithuanian climate experts. The participants answered a series of Likert scale questions, which included their perception of risk associated with climate change, emotional responses, and coping strategies. While it is possible that the use of Likert questions may have restricted the ability of participants to freely express their emotions, the researchers did note various emotional responses, and used factor analysis to distinguish ‘Morality-Based’ and ‘Consequence-Based’ responses. Morality-Based emotional responses included ‘other-related’ (e.g., indignation or contempt) and ‘self-related’ (e.g., guilt and shame) feelings. Consequence-Based emotional responses included ‘Retrospective’ (e.g., regret or sympathy) and ‘Prospective’ (e.g., worry or fear) feelings. These factors indicate the complexity of emotional responses for climate scientists, and the cognitive load this might involve. From a moral perspective, one can see both contempt for the failure of others, *and* shame for one’s own failings. Kaufman (1996, p.40) calls contempt ‘the affect of rejection’. If climate change is discussed in a way where there is a lack of respect between different sectors (scientists, policy makers, media), it risks impeding the progress of society in responding helpfully, because ‘contempt breeds contempt’ (Kaufman, 1996, p.39). Such morality-based responses suggest a perceived violation, by climate scientists, of ‘one’s own or others’ motivation or capacity to behave in a just and ethical manner’ (Drescher et al., 2011, p. 9). The psychological dysregulation resulting from not being able to act in line with moral decisions could lead to feelings of shame (‘self-related’), while witnessing others act in violation of moral decisions could breed feelings of contempt (‘other related’) (Jovarauskaite and Böhm, 2020; Morley et al., 2019).

#### Emotional distancing

Some climate scientists described ‘emotional distancing’ as their emotional response style. One participant, for example, stated ‘*it sometimes amazes me how much I actually know but how much I can put aside*’ (Renouf, 2021, p.11). It has been hypothesised that adopting this distance allows scientists to maintain a position of rationality (Head and Harada, 2017), and helps them to remain engaged in their work without being overwhelmed. Others, however, report feelings of acceptance that humanity has lost the battle to stop climate change (Renouf, 2021). Renouf (2021) questions whether this is better framed as ‘acceptance’, or as resignation/submission. The fact that such a cognitive shift appears to be linked to scientists finding a sense of ‘*inner peace*’ (Renouf, 2021, p.11), with less fighting and resisting the scientific realities. This distinction is important, as it fits well with the concept of ‘radical acceptance’ and reduced distress (Robbins et al., 2011) rather than the sense of hopelessness and increased distress that usually characterises resignation (Blalock and Joiner, 2000).

#### Hope

Hope was reported alongside the painful emotions and sense of inevitability. Duggan et al. (2021) reported that ‘Hopeful or Optimistic’ was noted 48 times in first time contributors to the ‘Is this How You Feel’ project, increasing to 71 by second time contributors. Additionally, ‘hope’ was the most common emotion expressed in Wang et al.’s (2018) study, noted 21 times. Renouf (2021) described how ‘hope’ appeared to be based on four factors in their interviews: (1) increasing social awareness about climate change, in particular amongst the youth; (2) awareness being seen as conducive to social change; (3) innovations and new technologies; and (4) faith in humanity’s ability to adapt. Duggan et al. (2021) paint a more complex picture of ‘hope’ involving two types: ‘Logic Based Hope’ and ‘Wishful Hope’. Logic based hope was associated with being able to notice specific, positive changes being made, and was thus linked to radical acceptance, and the notion of ‘active’ or ‘constructive’ hope which has been described elsewhere in the literature as an activating force (Macy and Johnstone, 2012; Ojala, 2012b). In contrast ‘Wishful Hope’ was associated with negative statements about the current state of the world in relation to climate change, and a desire for future change, rather than beliefs that such change is possible, or how this might come about. Logic based hope was reduced in second-time contributors, perhaps reflecting the aforementioned impact of long-term existence in the world of climate science and current global context. The importance of logic based, rational and active hope, was described as the ‘*small bright light at the end of a very long, dark tunnel*’ (Hayhoe, 2021, p.47), that is only possible to engage with when the gravity of our current situation is acknowledged.

### Intrapersonal processes at play in climate scientists’ experiences of climate change

Eleven of the papers discussed intrapersonal processes in climate scientists’ responses to working in, and contributing to, the debate around the climate crisis. These papers included secondary data analysis (*n* = 4), questionnaire or survey data (*n* = 2), interviews (*n* = 2) and personal reflective pieces (*n* = 3).

#### Compartmentalising

A commonality was the need to split into a professional and non-professional self, and the different responses climate science could elicit in each: ‘*weather patterns that delight me as a researcher chill my spine as a human being: I stare at the lines curving up and see the people who endure them*’ (Reay, 2018, p.303). Head and Harada (2017, p.38) described how this compartmentalising of the self into work and home life might protect the scientists from experiencing the more challenging aspects of their job constantly: ‘*I do not think I’d be in very good shape if I let myself think about it all the time*’.

Others described how their constant exposure to the scientific realities of climate change seemed to lead them to regard climate change as less personal, and with this, less distressing: ‘*I’ve thought for so long…that I do not personalise it to the extent that other people do*’ (Renouf, 2021, p.11). Whether this is an active, chosen strategy, or automatic habituation process, distancing work-life and personal-life in some way seemed to protect the wellbeing of some climate scientists.

#### Protective distancing

A type of denial was another mechanism scientists used to avoid the psychological paralysis that could arise from persistent engagement with the negative consequences of anthropogenic climate change. Head and Harada (2017, p. 38) note the protective nature of denial in climate scientists: ‘*we all know we are going to die but most of us who are reasonably socially well-adjusted do not think about it every day because it’s paralysing, and denialism is a form of protecting people against paralysis I think*’.

It is possible, here, to draw a parallel with Existential Psychotherapy. Becker (1973) notes the impossibility of living with a constant awareness of one’s own death, while acknowledging that this reality must be faced to live an authentic existence. What above was called denialism, might be better conceptualised as a healthy distancing from existential threat, which would allow for a re-approaching and re-engaging with that same threat at appropriate times. This healthy distancing was noted by Miner (2023, p.3) who noted that respite from the constant stream of information about the climate crisis allowed her to ‘*rededicate myself to continuing to fight for everything we can still save*’. Yalom (1980) explores Heidegger’s philosophy of ‘mindfulness of being’ where one notices *that* things are, rather than the *way* they are. In this mode of being, Yalom (1980) argues, one can embrace both the possibilities and the limits of one’s existence. Acknowledging the presence of the threat of climate change, and the finiteness of time and resources to address the causes of climate change, could invigorate and sustain climate scientists. As one becomes aware of the limits of a problem and one’s abilities to engage with it, one is also able to notice the possibilities for change and active positive engagement.

### Interpersonal processes at play in climate scientists’ experiences of climate change

Seventeen papers discussed interpersonal processes affecting climate scientists’ experiences of living with climate change.

#### Managing public attacks

Scrutiny and criticism from the media, politicians, and denialists were characterised as ‘*relentless*’ (Head and Harada, 2017, p. 37). ‘Graveyard humour’ was considered essential to cope with these regular attacks (Head and Harada, 2017; Reay, 2018). Such attacks were recognised as being aimed at undermining the work of climate scientists (Cologna and Siegrist, 2020), and as being largely motivated by people and corporations who wished to protect vested interests, be they ideological, financial or both (Campbell-Lendrum and Bertollini, 2010), and usually seeking to amplify their own interests (Beck, 2012). Examples were given of various strategies to seed mistrust in the science, such as in Australia, the then-Federal Treasurer, Scott Morrison, described opposition to Australia’s (high carbon emitting) energy strategy as ‘ideological [and] pathological’ while the Queensland Deputy Premier, Jeff Seeney, allegedly called climate change a ‘semi-religious belief’ (Tangney, 2018). These attempts to undermine the scientific method, and integrity of climate scientists, attack both the accuracy of the scientific consensus and the core beliefs and values held by this group. Such attacks may harm both individual scientists, and potentially fuel societal apprehension about endorsing the findings of scientists, while the role of necessary uncertainty in scientific modelling and prediction remains misunderstood.

Such attacks required a degree of stoicism, often times supported by other strategies such as ‘graveyard’ humour. Humour allows recognition of the profound threats of climate change in a less ‘emotive’ way was helpful and quite common (e.g., jokes such as ‘we are going to need a bigger boat’) (Reay, 2018). Without such strategies available, some thought it could preclude scientists from this field of work: ‘*if that sort of thing gets you down then you are no longer a climate scientist*’ (Head and Harada, 2017, p. 37). This places the climate scientist in a double bind. By the standards of their own discipline, they must remain stoic in the face of harsh criticism and attacks attempting to discredit or undermine their work, or they risk no longer being considered a climate scientist at all.

Where this has been a feature in the lives of other professionals (i.e., legal professionals), vilification has been associated with increased mental distress (O'Sullivan et al., 2022). The noted remedy was outspoken support from high authority figures, in this instance, from the Attorney General. For climate scientists, clear and supportive communication from institutions, media outlets, and political figures might provide a helpful remedy to the impact of vilification.

#### Duty as a scientist in a political arena

Climate scientists are in a painful position of being fully aware of the threats of climate change and the urgent societal transformations required, alongside seeing the failures of powerful bodies to act in line with the science. As scientists, they are expected to act as dispassionate experts, yet they are working in a field that increasingly involves significant political polarisation (Hayhoe, 2021). This tension was reported by some scientists who felt duty bound to shape public discourse and policy in the climate change debate, given the urgent nature of the problem, and the view that difficult choices need to be made quickly: ‘I *also consider it my duty as a scientist and as a citizen to try to inform the public and policy makers clearly about the predicament we are in*’ (Wang et al., 2018, p.29). Others regarded the role of a climate scientist as one where they must explain widely accepted scientific concepts without ‘*preaching*’ (Wang et al., 2018, p.28) to the public: ‘scientists *should produce quality research…not shout around*’ (Finnerty et al., 2024, p.3).

Gregersen and Bye (2023) and Tollefson (2021) reported that 81% of scientists in one IPCC survey believed that scientists *should* engage with advocacy relating to climate change, while only 66% actually do engage. Conversely, even a climate sceptic noted ‘*you cannot avoid [articulating feelings like worry or anxiety] …They are also human beings*’ (Nicolaisen, 2022, p. 677). Others noted that emotion adds a sense of reality to ‘*dry numbers and graphs*’ (Nicolaisen, 2022, p. 677). In Nicolaisen’s (2022) study, the need for neutrality was demanded of climate journalists, rather than climate scientists, as long as the emotions do not interfere with the research. Climate activism posed a similar discrepancy. While some viewed activism as a moral duty, others saw it as having the potential to damage credibility, and risk being labelled a ‘tree hugger’ (Finnerty et al., 2024). This discrepancy speaks to the urgent nature of the climate crisis, and the deep concern most climate scientists have about the future. They recognise that the role of scientists is to offer clear evidence that should be used to shape policy appropriately, but also recognise that societal narratives and media reporting often fail to reflect their findings accurately. This perceived violation was reflected in descriptions of mainstream media, seen by some as a vehicle for creating narratives of division between climate scientists that may not, in fact, exist: ‘*dissent (whether real or imagined) sells newspapers*’ (Spies, 2017, p. 51).

#### Media misrepresentation

When the media reports on climate science, it suggests that it should provide a balanced picture, where ‘balance’ has been interpreted by some as equating to giving the same airtime to opposing views (including those denying anthropogenic climate change). However, such an interpretation is at odds with the fact that 97% of scientists publishing on the issue of climate change offer clear evidence for anthropogenic climate change (Campbell-Lendrum and Bertollini, 2010). Thus ‘balance’ in the media is in fact a drastic misinterpretation of the scientific consensus. Other media strategies include ‘cherry picking’ of data by so called ‘climate-deniers’. Guo (2021) noted one example where Zhao et al.’s (2021) data were misrepresented and reported in a way that claimed that climate change would save lives. This claim gave a false account of the data, failing to account for mortality indirectly linked to climate change (e.g., floods, droughts, or pollution). Such media strategies risk shaping an inaccurate picture where climate scientists, and even the scientific process, are unfairly brought into disrepute (Spies, 2017).

One issue arising with media representations of climate change is discrepancies between how scientists feel they should respond. Climate science is based on the epistemology of the scientific method, of hypothesis testing, and the concept of falsification (Popper, 2002). In contrast, as discussed by Tangney (2018), those who attack scientists are more likely to base their views on ideology and personal interest (Campbell-Lendrum and Bertollini, 2010), rather than empirical data. If this becomes increasingly accepted by society, it is possible that scientists could feel ‘othered’ by, and alienated from, the wider society which they are both part of, and are working hard to protect. Jaspal et al. (2012, p. 401) explored reader comments in the Daily Mail, and noted one comment stating, ‘*if we could trust or believe these … money grabbing grant taking scientists*’. This sentiment has situated scientists as driven by money rather than empirical data, and as being different to the general public, in the same way that those with political power often are. Additionally, Cologna and Siegrist (2020) explored the role of trust in the scientist/public relationship. They reported that trust in environmental groups and scientists correlated strongly with climate friendly behaviours, and so it is important that the general public feel able to trust scientists. One way of increasing this sense of trust for scientific communication is to ensure that it is impartial and data-driven rather than persuasive and ideologically driven (Cologna and Siegrist, 2020).

#### Ways of engaging the public

There are different ways in which climate scientists may attempt to respond to media misrepresentation in terms of how they report their results or engage with the media. Some may hope to increase urgency and motivation for change in the public by reporting their findings ‘*in ever more catastrophic and apocalyptic terms*’ (Beck, 2012, p. 159). However, this risks a backlash from the general public, who may seek refuge in increasing levels of denial or an increasing sense of ‘climate fatigue’ as people switch off from the conversation completely (Beck, 2012). Nicolaisen’s (2022) reported that climate scientists (as well as climate journalists, and citizens) consider that the public ought to have a role as active participants in the climate science discourse, and suggest that this approach may engage the public in different ways and reduce the need for increasing apocalyptic reporting.

Aligned with this, Herman et al. (2017) analysed scientists’ contributions in print media, seeking to understand the rhetorical devices used by different ‘types’ of contributing scientist. Three types of scientists were identified (i.e., alerters, critics, and objectivists). Herman et al. (2017) noted potential variability between countries, as these data were based on Austrian media. It is also possible that there has been a shift in discourse and understanding since 2009 (the period of analysis), given the increase in prevalence of climate crisis related events. ‘Alerters’ were seen to represent IPCC consensus that climate change is anthropogenically caused, and strongly communicate warnings about the economic, political, and social causes, and consequences, of anthropogenic climate change. It is possible that ‘alerters’ may engage in this type of device because of their sense of duty or emotional responses to climate change (e.g., Renouf, 2021), but as yet the data does not offer a clear picture, and more work is required to understand what might differentiates ‘alerters’ from ‘critics’ or ‘objectivists’.

‘Critics’ portray climate science as ambiguous and seek to undermine the credibility of the scientific mainstream, while also suggesting competition or dissent within the group. This competition has the potential to undermine an important protective feature of a shared identity, and increase the psychological burden on climate scientists, as they feel ‘othered’ from their own in-group. For example, Light et al. (2021) explored articles written by consensus and anti-consensus scientists and found that anti-consensus authors are more likely to be located in North America, less likely to publish on the topic of ‘Forestry & Ecosystems’ or ‘Policy & Prediction’, and less likely to be connected to other authors through co-authorship. This suggests that anti-consensus scientists form a somewhat homogenous group, and one that seems to fulfil the role of ‘*merchants of doubt*’ (Light et al., 2021, p.9), seeking to delegitimise scientific consensus.

‘Objectivists’ acknowledge that evidence for anthropogenic climate change is compelling but understand climate researchers as a heterogeneous group (Herman et al., 2017). Adopting a middle ground, and a position considered by ‘Objectivists’ to be more pragmatic, has the potential to be positive *and* negative. The positive is that such an approach may be more amenable to media engagement and thus facilitate greater dissemination and explanation of climate science data. The negative is that this is burdensome, if it requires scientists to moderate their engagement and minimise their very real emotional disturbance, it may also lead to less accurate portrayal of climate risks.

### What mitigates and what exacerbates these experiences?

Climate scientists appeared to deploy a mixture of strategies to cope with the impact of their work, with both engagement and avoidance behaviours, dependent on context.

#### Mitigating climate emotions

Nine of the papers described how emotional responses to climate change might be mitigated and/or exacerbated. As described above, Head and Harada (2017) and Reay (2018) noted the protective roles of humour and finding pleasure or meaning in one’s work (e.g., as ‘cool’, or ‘fascinating’). Ojala (2012a) refers to this ‘turning towards’ as meaning-focused coping, where beliefs and values are drawn upon to sustain positive well-being, and has noted that children employed ‘trust’, ‘faith’, and ‘hope’ as examples of meaning-focused coping in the face of climate change. This was echoed by climate scientists, intimating the shared humanity of such experiences across generations (Renouf, 2021; Clayton, 2018; Duggan et al., 2021). Climate scientists used such strategies to remain engaged with the work: ‘*I got involved primarily in a science perspective on climate change because I felt that it was a really interesting science problem*’ (Head and Harada, 2017, p. 39).

#### Solution focused coping strategies

Studies indicated that few scientists currently engage in emotion-focused coping, for example by delving into climate related feelings in order to understand them and instead, they tended to endorse solution focussed approaches, concentrating on ways to help solve climate change (Jovarauskaite and Böhm, 2020): ‘the *best treatment for climate grief…is knowing you have made a contribution to reducing emissions or building resilience*’ (Miner, 2023, p.3). This included figuring out ways to ‘solve’ and/or ‘handle’ climate change, teaching others, and engaging in committed personal action to reduce one’s own carbon emissions to ‘net zero’ (Reay, 2018). Miner (2023) noted the need for scientists to move from education and advocacy, to providing solutions. Cologna and Siegrist (2020) report that having a low personal carbon footprint, allows climate scientists to conform to values of concern and care for the planet, which means that others are more likely to alter their own personal energy consumption. This may reflect a common assumption of modern science as being able to ‘solve’ problems and may also suggest how scientists perceive their role in society.

#### Increasing fear for future generations

Awareness of future generations, and the hypothesised reality that they will have to endure, was also noted as a stressor: ‘*I worry about the world I will be leaving my children and my grandchildren*’ (Renouf, 2021, p.9). Climate scientists are deeply aware of how future generations, and people living in the global south are being failed (Renouf, 2021), and some feel personally responsible: ‘*I do not want to become the generation that future children talk of as having destroyed the planet*’ (Wang et al., 2018, p.29). Such collective failure and regret indicates how climate scientists could see their own lives as involving acts of commission by having to live within sociopolitical structures which are damaging.

#### Unrealistic expectations about certainty and error

External factors could exacerbate these experiences, including the societal preference and even demand for ‘certainty’ before enacting drastic behavioural change (Kahan, 2010). Although the evidence for anthropogenic climate change, and the catastrophic consequences humanity faces, are areas of global scientific consensus, science itself cannot offer 100% certainty. This is further complicated by events like ‘Climategate’ where an admission of an error was met with accusations of scientific misconduct (Bodenhorn, 2013). If scientists are no longer allowed to factor in ‘uncertainty’ as a variable in their models, an entire epistemology is undermined. Any scientific model includes uncertainty. What is uncertain in this instance is not *that* climate change is happening, but the speed and degree of warming *at which* it is happening (Pörtner et al., 2022). For one climate scientist, the only thing keeping them awake at night was the possibility that their work was incorrect (Head and Harada, 2017), demonstrating the pressure climate scientists are feeling to ‘get it right’.

#### Perceived remoteness

A final external factor that might exacerbate the emotional burden of climate scientists is the perceived remoteness of an event. Renouf (2021) noted that 14 out of the 16 scientists reported feelings of personal vulnerability. The 14 scientists who reported feelings of vulnerability were the ones for whom the impacts of climate change were perceived as *less* remote, or more proximal, and/or suggestive of greater confidence in the adaptive abilities of their countries. This is, perhaps, a reflection of the closeness of the impacts of climate change on the global South, and how spatial proximity to the impacts of climate change leads to an increased emotional burden.

## Discussion

In scoping and reviewing existing literature on the psychological and emotional experiences of climate scientists, this paper has demonstrated that there are significant emotional impacts arising from working as a climate scientist, along with an associated set of coping strategies. Emotional distress in climate scientists appears to be a common and shared experience, one that involves both intra- and interpersonal processes, which highlights how people working in this profession may be vulnerable to experiencing distinct pressures and psychological burdens related to their work.

Distress, however, was only part of the storey, and in addition to the painful emotions reported by climate scientists (Duggan et al., 2021), there existed an excitement and ‘delight’ for climate scientists as they engage with the work itself (Head and Harada, 2017; Reay, 2018). The privilege of working in a professional domain (i.e., science) that is interesting and aligned with one’s values is likely to be a protective factor for climate scientists (Davis et al., 2015). Climate scientists may benefit from being in the position of some authority, working in a field that may align to their core values, both scientific and environmental. Having the opportunity to engage in meaningful climate action, which is proven to be one factor that associated with the amelioration of ecological distress in other populations (Gunasiri et al., 2022).

The findings in this review do not indicate specific mental health problems or associated maladaptive behaviours in this population, and there is no suggestion that their distress is a form of ‘mental illness’. Rather the results indicate that climate scientists experience an understandable psychological burden associated with their work, which includes emotional responses, emotional expression, and intra- and interpersonal processes. As discussed, there are clear links between psychological burden, particularly chronic stressors, and worse wellbeing and mental health. This review is important in highlighting how this Diathesis-Stress framework is relevant to health and wellbeing implications for this group. To understand this fully, further research is urgently required to better explore when and how painful emotional experiences may persist for climate scientists, and whether this contributes to more sustained and severe psychological distress. Duggan et al. (2021) report some early evidence of persistent distress, but further research is needed, particularly as climate change and its impacts are happening so quickly. The fact that some climate scientists may suppress emotional expression is also important, and the long-term benefits and disadvantages of such a strategy in this group should be investigated (Duggan et al., 2021; Head and Harada, 2017). There are also possible new initiatives which could offer alternatives, such as developing community-based spaces such as Climate Cafés; a facilitated, open, confidential group which anyone (including climate scientists) can attend, designed to be a safe space to explore and share psychological responses to climate change (Climate Psychology Alliance, 2020; Calabria and Marks, 2024).

### The psychological burdens of climate change

The emotion regulation strategies reported by climate scientists paint a picture of labour. Developing a ‘thick skin’, turning away from a lived reality, radically accepting a painful reality, and actively shielding loved ones from conversations about climate change (Head and Harada, 2017) require effort and commitment. The Diathesis-Stress Model posits that individuals have a threshold for stress that, if exceeded, can lead to the development of psychological distress (Arnau-Soler et al., 2019). Intensity and chronicity of stress can break through such thresholds and lead to mental health difficulties. The consistent effort required to employ these strategies can be regarded as a stressor likely to increase the vulnerability of climate scientists to experiencing psychological distress.

As such, an increase in distress in climate scientists is *not* because they are experiencing a new type of psychiatric illness termed ‘ecological distress’, requiring diagnosis or medicalisation, it is because they are being placed under increasing levels of stress. The chronic stress arising from their proximity to the reality of the severe threats of climate change through immersion in the world of climate science, coupled with government inaction, and negative societal responses to their findings. If this is not addressed there is a risk that this will impact the sustainability of the role of a climate scientist, who may face challenges in continuing under such stress, for example burn-out. This could have impacts on the broader scientific community, and even lead to a loss of engagement between climate scientists, action and public policy. In contrast, addressing each, or any of these factors could reduce their stress, distress and vulnerability to mental health difficulties. Such changes could happen on the global scale, for example, if governments acted urgently in line with climate science, or if societal narratives changed to recognise the gravity of the threats. The recent electoral victory in Mexico of a climate scientist becoming president could provide an important touchpoint in political conversations around climate change.

As Head and Harada (2017, p.38) note, the job itself, the thinking about climate change every day, would be ‘paralysing’, so climate scientists ‘*cannot think about it every second*’ (Renouf, 2021, p.11). They must still, however, think about it far more frequently than many others, particularly those fortunate enough to be living in countries that are (for now) relatively untouched by the effects of climate change. Society, and the planet, depend on climate science. It is a vital profession that helps us to understand what is happening to the climate, and what actions are required to protect life on Earth. If society and governments do not listen or respond appropriately, and as the effects of climate change grow, climate scientists (and those who listen to them) will be under increasing levels of stress, and the outcome is likely to be the increasing severity of emotional and psychological distress. This is consistent with Duggan et al.’s (2021) finding that instances of expressing painful emotions increased in second time contributors to the ‘Is This How You Feel?’ project.

Preston et al. (2022) note that cognitive reappraisal is a more adaptive coping mechanism than expressive suppression. Expressive suppression was noted to be a response-focused emotion regulation strategy, where one restricts emotional responses to a stressor. Expressive suppression has been shown to correlate with negative affect and depressed mood (Haga et al., 2009). As noted previously however, eco-distress is not a mental health disorder, but a rational reaction to an existential threat. As such, cognitive reappraisal may not provide an useful alternative, and acceptance based intervention might offer useful antecedent-focused emotion regulation strategies. Radical acceptance is central to therapeutic approaches such as Acceptance and Commitment Therapy (ACT), for example. When used in an intervention with carers of people with a terminal illness, Davis et al. (2015) reported that greater acceptance was associated with less psychological distress. In the face of an irreversible and insurmountable situation, radical acceptance is psychologically healthy and involves acknowledging ‘what is’ rather than wanting reality to be different, while still acting in line with your values (Davis et al., 2015). As such, radical acceptance would not lead to disengagement from climate action, but by shifting one away from passivity, resignation, or escape, it creates a space in which one can find new, adaptive ways to engage with the world as it is now. The energy that would be spent in fighting or resisting the painful reality and associated emotions is freed up to be used on other things, as reality is accepted and honoured, enabling engagement with action that makes a real difference. Similarly, Compassion Focused Therapy may be relevant, and Gilbert (2014) notes that connecting with the reality of suffering can be a motivator for taking responsibility and control, and making wise choices, rather than dwelling in suffering. This standpoint paradoxically reduces suffering and creates a space in which new hope, grounded in realistic appraisal, and action, can arise.

### In group/out group

This review highlights the tensions between occupying, at least, two concurrent identities: scientist and citizen. Such identities are associated with distinct, often conflicting, emotional responses (e.g., curiosity as a researcher, worry as a parent). Devereaux-Jennings and Hoffman (2021) call this the ‘Paradox of Objectivity and Passion’, where scientists are asked to dispassionately study a topic that involves pain and hardship for human, and more-than-human, life, including themselves.

The *Social Integration Theory* (Wakefield et al., 2017) posits that having multiple identities could be protective. Wakefield et al. (2017) noted the importance of multiple social connections, with more group identifications associated with increased ‘Satisfaction with Life’. The authors hypothesised that multiple group memberships can lead to a stronger sense of meaning and more support. However, as climate change can threaten many of these identities at once, this protection may lose effectiveness. Furthermore, as certain media and politicians undermine and divide the climate science community, conflict is engendered (Herman et al., 2017; Spies, 2017), and social group identification loses protective power. Findings thus suggest that feeling disunited or excluded from the ‘climate scientist’ group, could increase one’s emotional burden and vulnerability to mental ill health.

### Moral distress and moral injury

While the data are limited, and one should exercise caution when interpreting the findings, there is evidence here that moral distress and moral injury might offer a useful conceptualisation of the experiences of climate scientists. Acts of commission and omission by oneself, and by others, can be morally distressing and/or injurious. This review indicates how climate scientists may bear witness to, fail to prevent, and even perpetrate behaviours that ‘transgress deeply held moral beliefs and expectations’ (Litz et al., 2009, p.1). They see others ignore their warnings and in turn may feel compelled to suppress core aspects of themselves, such as their environmental identity (e.g., how they dress and talk) (Andrews et al., 2016) to increase their acceptability to others with the hope of increasing the acceptability and dissemination of their work. This simultaneously undermines protective self-identity (Verplanken et al., 2020) with potentially deeper consequences, such as shame about suppressing a moral code, or caring about nature.

The mixture of exposure to negative information about the future and insufficient action from policy makers and governments (The Lancet Planetary Health, 2021), will contribute to moral distress. Climate scientists work within the Western epistemological scientific framework (i.e., that beliefs should change when faced with sufficient evidence), but this is not reciprocated by the wider culture, where their findings meet with backlash and disbelief from media, government (Spies, 2017) and the public (Beck, 2012; Jaspal et al., 2012). This could be construed as a betrayal, which can contribute to moral distress and injury (e.g., Hickman et al., 2021). The Deficit Model of Science Communication (Tangney, 2018) describes how the power of communicating good science (Tollefson, 2021), is undermined by unhelpful discourse, or lack of forum for discourse, as the scientists’ values and integrity are attacked by media and political figures (Tangney, 2018).

Living in a society that encourages people into a ‘culture of uncare’ (Weintrobe, 2020) may also fuel a sense of moral distress, and the sense of individual ‘exceptionalism’ this encourages (i.e., the belief that ‘I am entitled to whatever I want’). This may explain why the warnings and evidence of climate science are dismissed or ignored, as it challenges the belief that global problems are not our individual responsibility, and so there is no need for individual or societal change.

Climate scientists have expressed indignation, contempt, and disappointment (Jovarauskaite and Böhm, 2020) at the ‘*mass effort to ignore, defer, deny, and lie*’ (Wang et al., 2018, p. 29) about a problem affecting us all, but particularly those in the poorest and most vulnerable nations, who have contributed least to the problem. In Renouf’s (2021) study, most climate scientists were from WEIRD countries, with a twofold risk; if such scientists experience climate change as less proximal, they may underestimate the immediacy of the threat (although this may change as extreme weather in the global North grows). Additionally, this could perpetuate the dominance of Western science, devaluing or dismissing indigenous knowledge.

In summary, climate scientists work in a world that at times may socially and politically undermine scientific knowledge to preserve economic and political ideals (Tangney, 2018). This can lead to the transgression of deeply held values by others, and sometimes even by themselves. For some climate scientists, this may lead them towards ‘climate perfectionism’; unachievable expectations of being beacons of pro-environmental behaviour that are impossible to live up to, and thus another source of distress.

### Limitations

The data available on the psychological and mental health impacts of climate change on climate scientists are largely focused on the emotional expression of climate scientists, with less exploration of specific psychological and physiological impacts. Similarly, as noted earlier, the current definition of eco-distress is broad. While this can make it difficult to operationally define eco-distress, for this review, it felt important to keep definitions broad to capture the wide range of psychological and emotional experiences present in climate scientists. There is clear evidence that ‘anger’, ‘sadness’, and ‘rage’ are present in climate scientists (Renouf, 2021), with less evidence regarding specific mental health and functional outcomes. Future studies would benefit from exploration into other areas associated with mental health difficulties such as sleep, appetite, and motivation.

Despite attempts for the term ‘climate scientist’ to capture a particular group, there was heterogeneity in how the term was applied in practise. While some papers restricted recruitment to scientists of earth sciences, physics, and maths (Head and Harada, 2017), others included social scientists and academics in the humanities (Jovarauskaite and Böhm, 2020). The term climate scientist is likely becoming increasingly broad, with the authors of this very paper potentially considered, by some, climate scientists. As this exploration of the psychological experiences of this group continues, a clear, operationalised definition is important. Similarly, situating this group in the present moment is essential. The data in this review ranges from 2010 to 2024. These two time points represent distinct situations with distinct pressures and context. From 2010 to 2024 there have been several climate movements (e.g., Fridays for Future or Extinction Rebellion). Future research might helpfully explore the impact of these on climate scientists’ distress.

This review is limited by the small number of climate scientists available in the data set, with three studies reporting on the same data set (Clayton, 2018; Duggan et al., 2021; Wang et al., 2018), partly due to the lack of studies in this area. This may mean the review is unable to offer a representative summary of the broader population of climate scientists, particularly when one considers the risk of bias, for example through self-selection. As Tollefson (2021) note, 60% of IPCC scientists did not take part in the survey as it dealt with opinions rather than science. To mitigate the limited data available, this scoping review included grey literature. No grey literature was identified in the search. Future reviews might benefit from developing and implementing a ‘grey search’ plan (Paez, 2017). This can both expand the data set from which conclusions can helpfully be made, including creating a more balanced view of the evidence. As it is not always peer-reviewed, however, grey literature should be interpreted with caution, and as such the findings of this scoping review point towards preliminary development of the area and the need for new areas of research.

This review was not pre-registered. Pre-registration was not mandatory at the time when the project was in development (i.e., 2021) but is has become standard practise in the past 2–3 years. Pre-registration is aimed at increasing transparency and reducing bias in research, and the lack of pre-registration in this project is acknowledged as a limitation.

This review focused only on climate scientists, and other environmental/sustainability professionals may experience similar strains, and so should perhaps also be included in future work. Furthermore, all studies reviewed came from WEIRD countries, with some having poor cultural validity (e.g., Jovarauskaite and Böhm, 2020; Herman et al., 2017). However, there was some diversity in other studies. Renouf (2021), for example, included participants from 12 countries including Botswana, Fiji, Germany, India, and the United States. Even so the limited variability of participants limits the conclusions as most reports come from climate scientists who live in parts of the world that are both most responsible for climate change, and most geographically and economically protected from its consequences. Future research must have larger samples and be more inclusive of experts in climate and ecology from across the world, including indigenous populations.

### Conclusion

There are specific emotional burdens on climate scientists, including the emotional responses, coping strategies employed, and the group dynamics at play. Future research would benefit from exploring the role of social connectedness within the climate science community, as well as the impact of values and acceptance based psychological interventions on supporting climate scientists to tolerate distress and feel able to remain engaged with the important work that they are doing. Future research would benefit from specific exploration of some of the process discussed here (i.e., social connectedness as a predictor of positive wellbeing or the impact of moral injury or moral distress on climate scientists).

Political discourse can be seen as a contributing factor to the mental health and wellbeing of the world’s climate scientists and if society is to support their vital work, discourse needs to shift away from the pursuit of ideological and economic interests in favour of coherent, honest, and respectful conversation about data. This should be echoed by media reporting that prioritises accurate dissemination of knowledge and responsible reporting rather than inflammatory reporting aimed at increasing readership. As citizens of the world, all of humanity has a responsibility, and an interest, in demanding these changes of our institutions.
