# Supplementary material for: A scoping review of the impact of eco-distress and coping with distress on the mental health experiences of climate scientists
Source: Front Psychol. 2024 Oct 21;15:1351428. doi: 10.3389/fpsyg.2024.1351428 (PMC11532191; doi:10.3389/fpsyg.2024.1351428)
Supplement: Supplementary file 1 [file Data_Sheet_1.PDF]

Appendix A: Charted Data

Authors/ Year	Methods		Participants			Outcome						Key Conclusions	Intrapersonal or Interpersonal	Blame or Responsibility
	Design	Study duration	Total number	Age	Sex	Survey outcomes	Frequency of emotional expressions	Changes in behaviour	Coping strategies	Qualitative outcomes	Lived experience			
Beck (2012)	Review of how the Intergovernmental Panel on Climate Change (IPCC) has dealt with increased public scrutiny since, so called, 'Climategate'	2010-2012	N/A	N/A	N/A	-	-	Advised to "keep a distance from the media"	-	-	-	In an attempt to capture public attention some scientists run the risk of portraying climate change in increasingly catastrophic and apocalyptic ways. The continuous alarming messaging could inspire backlash and 'Climate-fatigue'.	Interpersonal	Scepticism is manufactured by a "few motivated individuals" seeking to advance finances of corporate sponsors, and misrepresenting science as 'irrefutable'.<br><br>Undermining the authority of 'experts'
Bodenhorn (2013)	Review of the relationship between between evidence, modelling, and prediction.	-	N/A	N/A	N/A	-	-	-	-	-	-	A tension between the need for certainty and the acceptance of uncertainty.<br><br>In the example of 'Climategate' the admission of error led to accusations of scientific misconduct.	Interpersonal	There are social and political pressures to produce 'knowledge'. The reticence of society and political forces to tolerate uncertainty puts demands on scientist to act out of line with scientific values and epistemology.
Campbell-Lendrum and Bertollini (2010)	Editorial	-	N/A	N/A	N/A	-	-	-	-	-	-	A recent survey in the UK showed that only 26% of the public believe that climate change is happening and is man-made.<br><br>In the USA, 34% of people believe that most scientists think that global warming is happening.<br><br>Media coverage gives equal weight to extreme opposing positions. Lobbies seek to amplify evidence supporting their own interests, and attack opposing views.<br><br>Individuals ignore information suggesting that they should make unwelcome behavioural changes.	Interpersonal	Links to the interpersonal processes at play, as well as factors that might multiply negative experiences of climate scientists (i.e inaction on behalf of others in response to climate science findings and predictions).<br><br>Need for bi-partisan approach, rather than climate change being a political issue.

Authors (Year)	Methods		Participants			Outcome						Key Conclusions	Intrapersonal or Interpersonal	Blame or Responsibility
	Design	Study duration	Total number	Age	Sex	Survey outcomes	Frequency of emotional expressions	Changes in behaviour	Coping strategies	Qualitative outcomes	Lived experience			
Clayton (2018)	Secondary data analysis of frequency of emotions referenced in the 'is this how you feel' project	Project data collected between 2014 and 2018	43	Not stated	Not stated	Most commonly referenced emotions were 'frustrated', 'concerned', 'hopeful', and 'sad'.	Frustrated- 13 times Hopeful- 12 times Concerned- 11 times Sad- 9 times	Developing resilience	-	Participants commonly referred to feeling frustrated and concerned, but also hopeful.	-	Accepting a diversity of emotional responses among Climate Scientists without undermining their collective identity.<br><br>Need to develop coping strategies to protect climate scientists' mental health	Intrapersonal	-
Cologna & Siegrist (2020)	Meta-analysis	Literature search completed between September 2018 and January 2019	46 articles included	N/A	N/A	-	-	-	-	-	-	Trust in environmental groups and scientists corrected most strongly with climate friendly behaviours.	Interpersonal	Deliberate and organised attempts to undermine scientific consensus in climate science, propagating mistrust.
Duggan et al. (2021)	Secondary data analysis of frequency of emotions referenced in the 'is this how you feel' project	Project data collected between 2014 and 2020	73 letters	Not stated	Not stated	Most commonly referenced emotions were 'afraid', 'angry', 'hopeful or optimistic', and 'sad'.<br><br>Distress and burnout noted in the context of climate anxiety and climate grief.<br><br>Difference between 'logic based hope' and 'wishful hope'. 'Logic based hope' was much reduced in second time contributors.	Afraid- 69 times in first time contributors, 92 in second time contributors.<br><br>Angry- 49 times in first time contributors, 61 in second time contributors.<br><br>Hopefully or Optimistic- 48 times in first time contributors, 71 in second time contributors.	-	-	Participants commonly referred to feeling frustrated, guilty, determined, faithful, hopeless, outraged, and powerless.	-	Normalise emotional responses to climate change among climate scientist.<br><br>Need for Climate Scientists to be supported emotionally and feel able to continue their work.<br><br>Importance of sense of community within climate scientists. Opportunity to express emotions and to not feel alone in their feelings.	Both	-

Authors (Year)	*Methods*		*Participants*			*Outcome*						*Key Conclusions*	*Intrapersonal or Interpersonal*	*Blame or Responsibility*
	Design	*Study duration*	*Total number*	*Age*	*Sex*	*Survey outcomes*	*Frequency of emotional expressions*	*Changes in behaviour*	*Coping strategies*	*Qualitative outcomes*	*Lived experience*			
Finnerty et al. (2024)	Mixed methods design across a number of countries. Aimed at exploring association between scientist identity and science-activism relationship	Between 12/02/22 and 01/10/22	329 scientists from 41 countries	Mean age= 40.11 years	Female = 541.%<br><br>Male= 40.7%<br><br>Non-binary= 2.4%<br><br>Prefer not to say= 2.7%	Scientists should produce good quality research rather than engage in activism<br><br>Vs<br><br>Activism as a moral duty<br><br>Supporting rather than participating in activism.	-	-	-	-	-	The reputation of scientists is contended. Come feel like activism is an important duty while others feel this is out of remit for scientists.	Both	-
Hayhoe (2021)	Secondary data analysis of frequency of emotions referenced in the 'is this how you feel' project	Project data collected between 2014 and 2018	43	Not stated	Not stated	Most commonly referenced emotions were 'frustrated', 'concerned', 'hopeful', and 'sad'.	Frustrated- 13 times Hopeful- 12 times Concerned- 11 times Sad- 9 times	Developing resilience	-	Participants commonly referred to feeling frustrated and concerned, but also hopeful.	-	Accepting a diversity of emotional responses among Climate Scientists without undermining their collective identity.<br><br>Need to develop coping strategies to protect climate scientists' mental health	Intrapersonal	-
Head and Harrada (2017)	Interviews conducted with climate scientists	June-October 2014	13	"Ages ranged from the early career stage (defined in Australia as within five years of PhD completion) to retired but still academically active"	Female =4, Male=9	Common themes were identified regarding the cause of the emotional responses, an explanation of them, and a response to them/strategy to cope.<br><br>Emotions including anxiety, fear, and loss were noted.	-	The use of denial as a means of self-preservation.<br><br>Engaging in daily activities that can distract from the climate crisis.<br><br>Developing strict work life boundaries.<br><br>Avoiding informing children, which was considered a form of good parenting.	"graveyard humour"<br><br>"develop a thick skin"<br><br>Separation of work and family<br><br>Denial<br><br>Remaining optimistic<br><br>"We've just got to get on with it"	-	"I can't do it [be a climate scientist] 100% of the time"<br><br>Split between enjoying the work, and struggling with the bleak predictions	Need for laborious emotional regulation strategies in order to maintain their "credibility" as dispassionate scientists: "the heart is kept a long way from the brain"<br><br>Painful emotions are minimised, and pleasurable ones are maximised.<br><br>Social content of continued debate around climate denialism and 'truth' of anthropogenic climate change	Both	The possibility that the data and predictions are wrong<br><br>Being accused of being a fraud-hate male and death threats (not something that other scientists have to deal with)

Authors (Year)	*Methods*		*Participants*			*Outcome*						Key Conclusions	Intrapersonal or Interpersonal	Blame or Responsibility
	Design	*Study duration*	*Total number*	*Age*	*Sex*	*Survey outcomes*	*Frequency of emotional expressions*	*Changes in behaviour*	*Coping strategies*	*Qualitative outcomes*	*Lived experience*			
Herman et al. (2017)	Newspaper articles contributed to by climate scientists were analysed. Coding and interpretation of information, and 'rhetorical devices' was used to ascertain what "type" of scientist was contributing (i.e. alerter, critic, objectivist)	1 year (2009)	"34 different researchers presented their perspectives on climate change issues in the analyzed interviews and guest commentaries."	Not stated	Not stated	Three types of scientist identified (i.e. alerters, critics, objectivists).<br><br>Alerters: "represent the IPCC consensus on anthropogenic climate change and strongly warn about its consequences."<br><br>Critics: "Critics present climate researchers as a heterogeneous group and portray scientific knowledge as ambiguous and not compelling"<br><br>Objectivists: "Objectivists present climate researchers as a heterogeneous group with different perspectives on climate change. However, objectivists consider the scientific knowledge on anthropogenic and natural causes of cli-mate change basically clear and sufficiently compelling"	Alerter- 23<br>Critic- 6<br>Objectivist- 9	-	-	-	-	Types of climate scientist not equally represented, despite media efforts to give equal weight and air time to each voice.<br><br>Objectivists deliberately try to convey neutrality and an objective position<br><br>"our analysis points to a strong politicization of climate science in Austrian media coverage. All three types of researchers invoke scientific authority to position themselves politically and provide specific recommendations for action or inaction"	Interpersonal	Perceived in-group challenges to, what was believed to be, shared epistemic values<br><br>Journalists hand selecting particular scientists to express a predefined narrative.<br><br>Journalists seeking simple concrete answers too complex and abstract problems.
Jaspal et al. (2012)	Discourse analysis of reader comments on articles pertaining to climate science and/or climate scientists	Comments from 1.1.2010 to 31.13.2010	1907 reader comments included in the analysis	Not stated	Not stated	-	-	-	-	-	-	Readers used rhetorical strategies such as denigration, delegitimisation, and constructing a financially driven agenda of climate science to criticise climate science and climate scientists.	Interpersonal	-

	Methods		***Participants***			***Outcome***								
Authors (Year)	*Design*	*Study duration*	*Total number*	*Age*	*Sex*	*Survey outcomes*	*Frequency of emotional expressions*	*Changes in behaviour*	*Coping strategies*	*Qualitative outcomes*	*Lived experience*	***Key Conclusions***	***Intrapersonal or Interpersonal***	***Blame or Responsibility***
Jovarauskaite and Bohm (2020)	Participants completed online questionnaires regarding their views and opinions about climate change.	July-October 2017	215	Participants' ages ranged from 20 to 68 years. Mean age= 41.74	62.3% female 37.7% male	Fifteen items yielded four dimensions: Dreadful Consequences, Morality, Controllability, and Societally Disputed Risk<br><br>Morality based emotional responses: other related (indignation, contempt, disappointment), self related (guilt shame)<br><br>Consequence based emotional responses: retrospective (regret, sadness, sympathy), prospective (hopelessness, worry, fear).	Most frequent emotions noted were indignation, disappointment, sadness, and regret.	-	Problem focused coping (concentrate on solution, talk to others, how to handle climate change)<br><br>Emotion focused coping (delve into and understand climate related feelings, ask colleagues about how they cope, tell others how I feel, get advice from others)<br><br>Avoidance (distract myself, refuse to believe it is happening, pretend it isn't happening, avoid thinking about it)	-	-	In the cluster of emotionally engaged climate experts, there were 103 women and 48 men; The cluster of emotionally disengaged experts contained 33 women and 31 men.<br><br>More emotional engagement was associated with higher scores on problem- and emotion-focused coping strategies.<br><br>The odds of being emotionally engaged (vs. disengaged) regarding climate change were 3.80 times higher if climate experts worked in the government sector than if they worked in academia.	Intrapersonal	-
Light et al. (2021)	Text analysis of anti-consensus and consensus climate scientists	Papers published between 1970 and 2008	7354 articles written by 57 anti-consensus scientists, and 270 consensus scientists.	Not stated	Not stated	-	-	-	-	-	-	Non-consensus authors are more likely to be located in North America, less likely to publish on the topic of Forestry & Ecosystems or Policy & Prediction. They are also less likely to be connected to other authors through co-authorship	-	-

Authors (Year)	*Methods*		*Participants*			*Outcome*						*Key Conclusions*	*Intrapersonal or Interpersonal*	*Blame or Responsibility*
	Design	*Study duration*	*Total number*	*Age*	*Sex*	*Survey outcomes*	*Frequency of emotional expressions*	*Changes in behaviour*	*Coping strategies*	*Qualitative outcomes*	*Lived experience*			
Miner (2023)	Personal reflection on her own lived experience of climate change as a climate scientist	2023	N/A	N/A	N/A	-	Overwork, Exhaustion, extreme stress<br><br>Climate grief	Started taking weekends off<br><br>More time outside.<br><br>Re-wiliding	Grounding<br><br>Talking to colleagues<br><br>Work on solutions	-	-	Important to connect with natural world and fortify oneself so that one can “rededicate” oneself to continuing to fight	Both	-
Nicolaisen (2022)	Focus group exploring the perceptions on the roles of climate scientists, climate journalists, and citizens	Sept.-Nov. 2021	15 focus groups with 26 Danish climate scientists, 24 climate journalists, and 26 citizens	20-69	Female =27, Male= 49	Climate scientists have a responsibility to help journalists understand the complexities of climate change.<br><br>Climate scientists ought to communicate	-	-	-	-	-	Climate scientists have a right to express emotions.<br><br>It is their duty and responsibility to communicate	Interpersonal	-
Reay (2018)	Personal reflection on his own lived experience of climate change as a climate scientist	-	N/A	N/A	N/A	-	-	Buying 28 hectares of Scottish woodland to care for and use in a green way (?living in line with green identity)<br><br>Spending money on ecological tools (e.g. solar panels, or electric cars).	“gallows humour”<br><br>“wrestle back an iota of control” with personal behaviour changes<br><br>“for me the most powerful response is to teach”	-	“weather patterns that delight me as a researcher chill my spine as a human being: I stare at the lines curving up and see the people who endure them”<br><br>Gallows humour	Individual actions are important (e.g. buying solar panels), but it is necessary that they are done globally. For important, global, and sustained behaviour change, education is key.	Intrapersonal	-

	Methods		*Participants*			*Outcome*								
Authors (Year)	*Design*	*Study duration*	*Total number*	*Age*	*Sex*	*Survey outcomes*	*Frequency of emotional expressions*	*Changes in behaviour*	*Coping strategies*	*Qualitative outcomes*	*Lived experience*	***Key Conclusions***	***Intrapersonal or Interpersonal***	***Blame or Responsibility***
Renouf (2021)	This is the first stage of a wider research project.<br><br>This part of the research draws on 16 qualitative interviews with climate scientists to explore the meaning that climate scientists give to climate change through their lived experience	February to March 2019	Thirteen of the 16 participants had contributed to IPCC reports.<br><br>Years of experience working in area of climate change ranged from 10 to 30.<br><br>Participants were located in 12 different countries including Australia, Botswana, Canada, Fiji, Germany, India, Japan, Norway, Pakistan, Switzerland, the UK and the USA	Not stated	Eight of the participants are women and eight are men and all but two are parents.	“No one believed that the temperature increase would remain under the 1.5 °C threshold.”<br><br>The impacts of climate change would be felt, primarily, by younger generations.<br><br>The impact of climate change on agriculture, and the knock on effects of that would include food crises, climate migration, and “financial breakdown”.<br><br>Hope:<br>1. Increasing social awareness about climate change, in particular among the youth.<br>2. Awareness was seen as conducive to social changes.<br>3. Innovations and new technologies offered promise<br>4. Faith in humanity’s ability to adapt	Emotions (from more frequent to less frequent): angry, sad, rage, emotional distance, acceptance, hopeful, feeling of submission.<br><br>Inabilityto identify a single over arching emotion.<br><br>Some expressed exclusively painful emotions (e.g. anxiety, rage, or sadness).<br><br>Other, consciously or unconsciously, adopted an emotional distance with the topic.<br><br>One participant expressed “acceptance”<br><br>Reported feelings of hope only occurred spontaneously on three occasions.	Doing one’s “best”: research, teaching, public speeches; supporting work of a wildfire agency; and honesty with students.	Avoid personalising the understood consequences of climate change<br><br>Submission and acceptance of negative consequences of climate change.	-	Worry about the world being left for their children<br><br>Feelings of personal vulnerability<br><br>“Sometimes fear, sometimes a kind of determination to deal with it, and sometimes sadness as well”.	Temp unlikely to remain under 2.0c<br><br>Climate change associated with a number of concerns<br><br>Wide consensus that Climate change is disrupting normal ways of life<br><br>Impact of climate change experience as well as studied<br><br>Concern for indirect as well as personal impacts<br><br>Climate change is often relational and contextual<br><br>Emotional burden of lived experience<br><br>Hope	Both	Themselves and previous generations

Authors (Year)	Methods		Participants			Outcome						Key Conclusions	Intrapersonal or Interpersonal	Blame or Responsibility
	Design	*Study duration*	*Total number*	*Age*	*Sex*	*Survey outcomes*	*Frequency of emotional expressions*	*Changes in behaviour*	*Coping strategies*	*Qualitative outcomes*	*Lived experience*			
Spies (2017)	Review attempting to unravel the science from the politics, describe typical emotional responses, and discuss the importance of, and barriers to, achieving an international agreement on reducing emissions.	-	N/A	N/A	N/A	-	-	-	-	-	Cynical, resigned, annoyed about the problem.	“A psychologist could barely dream up a better scenario for paralysis than climate change” (Halstead, 2014).<br><br>Dissent (whether real or imagined) sells newspapers. Additionally, scientific dialogue is misrepresented as debate, bringing the validity of findings into disrepute.<br><br>So called ‘climate-deniers’ cherry-pick data and statements that support their views.	Interpersonal	An attack on science and/or scientist may well be the strategy of those opposing government action.<br><br>Blame companies and politicians seeking to maintain vested interests.
Tangney (2018)	This paper uses a review methodology to examine climate experts’ rhetorical tactics through the eyes of conservative policymakers	-	N/A	N/A	N/A	-	-	-	-	-	-	Deficit Model of Science Communication:<br><br>1. Assumption of public understandings<br>2. Assumption that understanding, guarantees favorable attitudes toward science<br>3. Assumption that any failing in communicating science arise from a failure on the part of the recipients.<br><br>Sceptics will reject scientific prediction in favour of pre-formed world views. Where science fails to reach a consensus, sceptics ,are accusations of a covert ideology.	Interpersonal	Where climate science is used for agenda-setting, there appears to be a poverty of scrutiny about the specific findings.<br><br>Political figures (e.g. then-Federal Treasurer Scott Morrison) make accusations of ideology influence scientific finds over data.<br><br>Former Queensland Deputy Premier Jeff Seeney described climate change as a “semi-religious belief”.<br><br>Attacks on the values and integrity of scientists, as well as undermining scientific epistemology.

Authors (Year)	Methods		Participants			Outcome						Key Conclusions	Intrapersonal or Interpersonal	Blame or Responsibility
	Design	*Study duration*	*Total number*	*Age*	*Sex*	*Survey outcomes*	*Frequency of emotional expressions*	*Changes in behaviour*	*Coping strategies*	*Qualitative outcomes*	*Lived experience*			
Tollefson (2021)	Feature article, analysing IPCC survey data	2021	92 out 233 scientists responded	Not stated	Not stated	61% of climate scientists report feeling “anxiety, grief, or other distress” as a result of climate change.	Most scientists claimed they experienced anxiety, grief or other distress (21%-frequently, 40%-infrequently).	81% believed that scientists *should* engage with advocacy related to climate change, and 66% *do*. This adovacy included promotion of science through publications (98%), dialogue with policy makers (40%), and participating in demonstrations (25%).<br><br>Climate scientists report concerns about climate change impact where they choose to live (41%), lifestyle choices, (21%), and whether or not to have children (17%).	-	-	-	Beliefs and hope in the power of communicating good science “by focusing on the best available scientific information it [the IPCC] has avoided the politicisation that has occurred with other scientific issues, such as masking and vaccinations for COVID-19”<br><br>A sense that nowhere feels safe, geographically: “so the question is, where do you go?”	Both	Government: “Governments are just at the stage of providing green promises, but so far we have not seen any action”<br><br>IPCC: “I want the IPPC to be more aggressive on that [engaging policy makers]”

Authors (Year)	Methods		Participants			Outcome						Key Conclusions	Intrapersonal or Interpersonal	Blame or Responsibility
	Design	*Study duration*	*Total number*	*Age*	*Sex*	*Survey outcomes*	*Frequency of emotional expressions*	*Changes in behaviour*	*Coping strategies*	*Qualitative outcomes*	*Lived experience*			
Wang et al. (2019)- study 1	The first study examined responses from climate scientists using data collected externally by the ITHYF project, in addition to responses from a new survey conducted by the current authors, asking students to report their feelings about climate change.	2018	Scientist data (n=44)<br><br>Students (n=94)	mean age= 20	Information not available for climate scientists, but 60% of the students were female.	Scientists referred to a multiplicity of emotions, including conflict: e.g. ""I feel nervous. I get worried and anxious, but also a little curious. The curiosity is a strange, paradoxical feeling that I sometimes feel guilty about"<br><br>"What motivated me to become a scientist in the first place was my desire to explain and model things we did not understand."<br><br>"It was never about preaching to others about an existing scientific consensus. I feel uncomfortable in this strange role nearly as much as I am in discussing about believing or not in climate change"<br><br>Vs.<br><br>"I also consider it my duty as a scientist and as a citizen to try to inform the public and policy makers clearly about the predicament we are in and the choices we cannot avoid"	Hope- (n=21)<br><br>Frustration- (n=16)<br><br>Responsibilit y- (n=13)	-	-	Climate scientists have an awareness of the situation. There is a sense of concern, and a mixture of emotions. There is also a sense of uncertainty about which steps to undertake to reduce CO2	-	Narrative of loss and responsibility<br><br>A number of emotions expressed ranging from hope, to anger, and frustration<br><br>Climate scientists were more likely to express thoughts about climate change relating to other areas and the things affected by climate change (children, the planet, humanity).<br><br>The bredth of self-identities among climate scientists meant that emotional responses were more widely connected. This connectivity also led to conflicting emotions as scientists (curiosity) or as parents (worry, fear)."	Both	-
